# COVARIATE-ADJUSTED ASSOCIATIONS OF COGNITION, DEPRESSIVE SYMPTOMS, AND SOCIAL SUPPORT WITH LIFE SPACE MOBILITY

**DOI:** 10.1093/geroni/igad104.2132

**Published:** 2023-12-21

**Authors:** Henrietta Armah, Olivio Clay

**Affiliations:** University of Alabama at Birmingham, Birmingham, Alabama, United States; University of Alabama at Birmingham, Birmingham, Alabama, United States

## Abstract

Diabetes mellitus is one of the most common chronic diseases among older adults and can affect their movement, participation, and all aspects of daily living, thus restricting life space mobility. Life space mobility reflects individuals’ ability to move independently and engage in social activities. This study examines covariate-adjusted associations of cognition, depressive symptoms, amount of support received and satisfaction with support with life space mobility. The study included 247 older adults aged 65 and above from the University of Alabama at Birmingham (UAB) Diabetes and Aging Study of Health (DASH). Average age was 73, 45% of the sample were Black/African American, 53% were female, and 47% were married. Results from multiple covariate-adjusted regression analyses revealed that being Black/African American, older, female, and higher depressive symptoms significantly predicted lower life space mobility (all p’s < .05) while being married, educated, and reporting better health significantly predicted greater life space mobility. Similarly, higher cognitive function was a significant predictor of greater life space mobility (B = .140, p < .05). Results remained significant even when adjusted for covariates. Amount of support received and satisfaction with support did not predict life space mobility. Findings from this investigation identify individuals who are at risk for restricted life space mobility and suggest protective factors. Establishing these associations with life space mobility within a health disparities framework would be important as it would draw attention to functioning in later life for socially disadvantaged groups and help inform interventions.

